# The promising fNIRS: Uncovering the function of prefrontal working memory networks based on multi-cognitive tasks

**DOI:** 10.3389/fpsyt.2022.985076

**Published:** 2022-10-25

**Authors:** Yufei Ren, Gang Cui, Xiaoqian Zhang, Kun Feng, Chenchao Yu, Pozi Liu

**Affiliations:** ^1^Department of Foreign Languages and Literatures, Tsinghua University, Beijing, China; ^2^Department of Psychiatry, Yuquan Hospital, Tsinghua University, Beijing, China; ^3^Independent Practitioner, Beijing, China; ^4^School of Clinical Medicine, Tsinghua University, Beijing, China

**Keywords:** fNIRS, prefrontal cortex, working memory network, neurovascular coupling, psychiatric disorders

## Abstract

The diversity of cognitive task paradigms using functional near-infrared spectroscopy (fNIRS) and the lack of theoretical explanations for these functional imaging atlases have greatly hindered the application of fNIRS in psychiatry. The fNIRS brain imaging based on multiple cognitive tasks could generally reflect the working patterns and neurovascular coupling changes in the prefrontal working memory network. By alternating the stimulation patterns of resting and task states, six typical symptom-related functional brain imaging waveforms related to psychiatric disorders are identified and three joint networks of the prefrontal working memory, namely, the attentional working memory primary coordination network, the perceptual content working memory secondary network, and the emotional-behavioral working memory executive network, are initially represented. This is the first attempt to characterize the cognitive, emotional, and behavioral regulation of the prefrontal working memory network using fNIRS, which may promote the application of fNIRS in clinical settings.

## fNIRS in psychiatry

The improvement of diagnosis and treatment of psychiatric disorders relies on accessible neuroimaging tools that can identify associated frontal lobe dysfunction. Functional near-infrared spectroscopy (fNIRS) is an emerging functional neuroimaging method, non-invasively using the absorption and scattering relationships between multiple wavelengths of near-infrared light and chromophores in brain tissue [see (1) for more technical details]. It detects the brain activity near the brain surface by monitoring the relative concentration changes in oxy-hemoglobin [oxy-Hb] and deoxy-hemoglobin [deoxy-Hb] in the microvasculature of brain tissue during cognitive activity in real-time (2, 3).

During neural activities, consumed oxygen is compensated by increased blood supply, leading to an increased [oxy-Hb] and decreased [deoxy-Hb], a process understood as part of the neurovascular coupling mechanism (4, 5). Neurovascular coupling is the basis for functional brain imaging (6, 7). As early as 1890, Roy and Sherrington proposed the theory of neurovascular coupling that is “an increase in cerebral blood flow is induced during neural activity for a better energy supply”; thus, the change in local oxygenated hemoglobin will cause a series of other reactions. Changes in [oxy-Hb] and [deoxy-Hb] are often associated with neuronal activity in specific regions related to distinct human functions (8); thus, it is capable of real-time acquisition of cerebral oxygen signals as well as dynamic monitoring of physiological and pathological processes in the cerebral cortex.

Although NIRS can only observe the functional activity of about 2 cm of cerebral cortex under the probe, compared with PET, single-photon emission computed tomography (SPECT), fMRI, and other neurological effect technologies, it is relatively mobile, concise, and easy to meet measurement conditions, allowing subjects to be tested in a more natural and comfortable environment. As a result, more realistic data are obtained and could be used by psychiatrists. In recent years, several studies have shown that patients with psychiatric disorders have impaired function in the prefrontal cortex [e.g., (9, 10)]. The fluctuation differences of the [oxy-Hb] and [deoxy-Hb] among different regions of the prefrontal cortex can characterize different types of mental disorders (11–17). To some degree, the functions of the prefrontal cortex could reflect the working patterns of working memory networks (18). However, the representation mechanism of the prefrontal working memory network reflected by fNIRS imaging is still not clear.

The fNIRS, in conjunction with the verbal fluency task, holds promise for understanding, detecting, and differentiating psychiatric disorders, and has the potential for developing accessible neuroimaging biomarkers for different psychiatric disorders (19). The multi-site study (13) is the first large-scale, case-control study that demonstrates the utility of NIRS to differentiate the diagnosis of major psychiatric disorders in natural clinical settings using verbal fluency tasks.

However, in China, it is generally found in clinical psychiatry that the current diagnostic criteria developed in Japan for unipolar depression and bipolar depression are of low diagnostic compliance in clinical application. Due to the low diagnostic compliance, the application of fNIRS in psychiatry is constrained. The low diagnostic compliance may be related to factors as follows: (1) there is only a single diagnostic reference imaging of bipolar and unipolar depression. It does not take into account the dynamic feature of the disease and the states of patients. With the remission of the disease, changes in brain functioning have an impact on the corresponding cerebral blood flow diagram. Some studies show that patients with depression show non-dominant hemisphere activation in thinking during depressive states and with their recovery from depression, a preference is returned to the dominant hemisphere in thinking (20). Besides, the change in a particular brain region or waveform is unstable and variable due to the patient's state. Schecklmann et al. (21) suggest that the analysis of brain waveforms in groups or strings is more valuable and reproducible. (2) Different cognitive tasks and modalities should be incorporated as the stimulation covers a wider and various brain regions. In fact, cognitive resources required for various tasks are different, which might lead to confusion with slight changes in task requirements. (3) Only the change in [oxy-Hb] is considered, but not [deoxy-Hb]. Culver et al. (22) reported that the sum of [oxy-Hb] and [deoxy-Hb] concentration is more advantageous in the near-infrared cerebral blood flow topographic images, and is more suitable for large samples and long-term stimulation mode. (4) Language typological differences between Chinese and Japanese may cause differences in the same language task such as verbal fluency task, let alone among different cognitive tasks with varied difficulties. Therefore, it is crucial to find a suitable clinical task in China that can reflect the dynamic changes in patients' status based on energy coupling mechanisms.

## Multi-cognitive tasks paradigm

Multi-cognitive tasks include four cognitive tasks, namely the emotional picture identification task, (category) verbal fluency task, finger-tapping task, and setting-based sentence fluency task. The alternation of adaptive tasks and primary tasks as well as the alternation of resting and task states ensures the various representations of the prefrontal cortex in its cognitive regulation and cognitive dysfunction. A detailed introduction of tasks involved in the multi-cognitive tasks' paradigm is shown in Table 1. The involved tasks exhibit theoretical significance. Most fNIRS studies on speech production have employed the verbal fluency task in patients with psychiatric disorders (21, 23), whereas setting-based sentence fluency task is adapted from visual picture naming [see (24, 25)], which has been scarcely studied in fNIRS. As for the adaptive tasks, the emotional identification task is essential in studies of emotions [see (26) for a review on emotion perception]. Finger-tapping tasks are one of the most common paradigms used to study the human motor system in functional neuroimaging studies [see (27) for a review]. Our multi-cognitive tasks paradigm combined these tasks in adaption to induce greater activation of prefrontal working memory networks as well as emotion regulation, taking the alternation of task properties and of resting and task states into consideration for the concern about patients.

**Table 1:** Detailed introduction of the multi-cognitive tasks.

**Multi-cognitive tasks**	**Task property**	**Task requirement**	**Task timing**
1. Emotional picture identification	Adaptive task	Identify whether pictures are positive/negative/neutral	15 pictures presented 4 s with a 2-s button response respectively (total: 1.5 mins)
2. (Category) Verbal fluency task [e.g., (15–17)]	Main language task	Categorize the hyponymy of a given category	Four category neutral words with 30-second-response and 30-second-rest respectively (total: 4 mins)
3. Finger tapping task	Adaptive task	Imitate the finger movement shown on the computer screen	Each hand imitates the given movements for 30 s with a 30-second-interval (total: 2 mins)
4. Setting-based sentence fluency task	Main language task	Produce sentences based on the given negative pictures	4 negative pictures with 30-second-production and 30-second-rest respectively (total: 4 mins)

**Figure 1:**
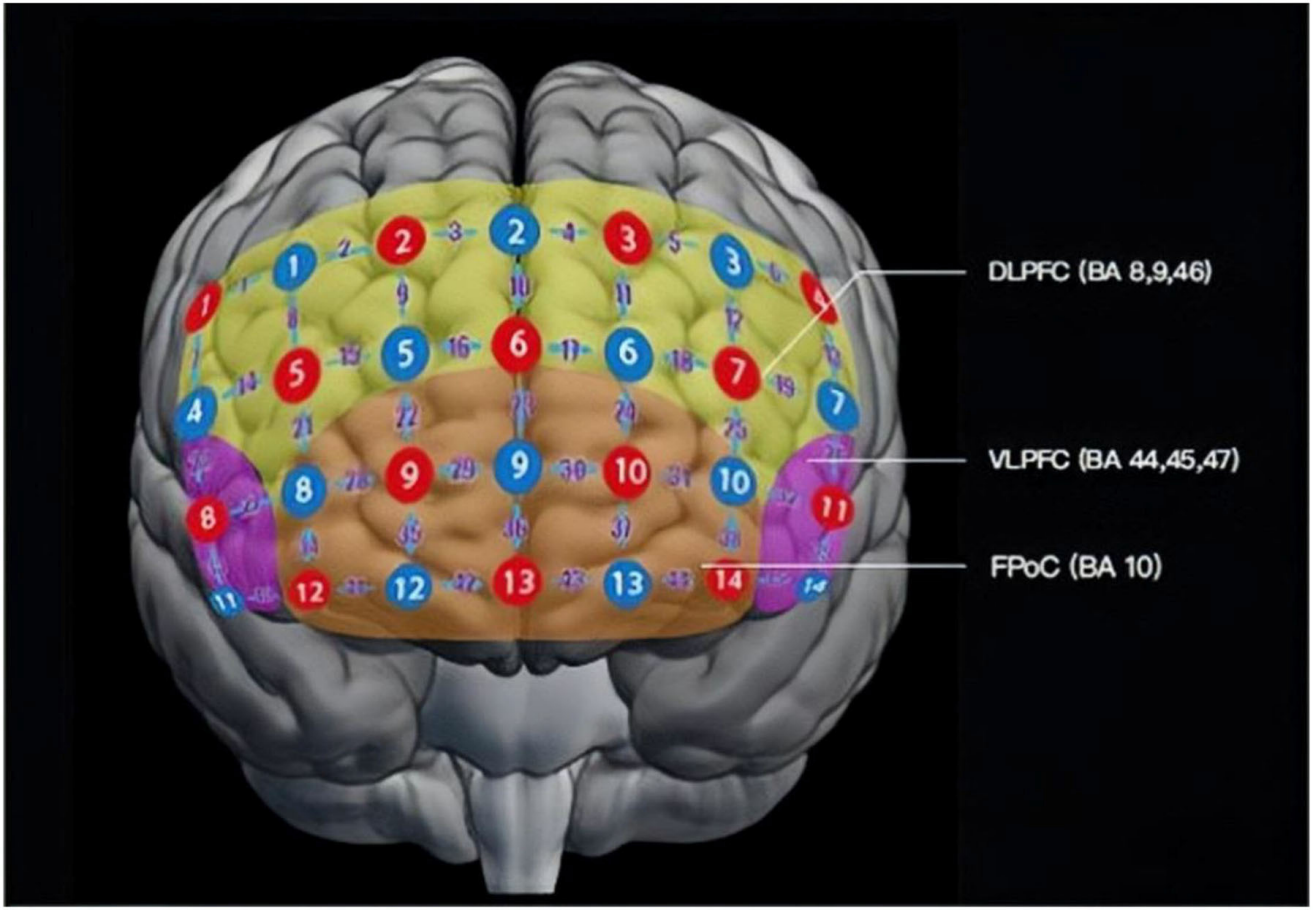
Layout of probes of the fNIRS probe in a frontal view. There are 14 emission probes (red) and 14 detector probes (blue) placed on the forehead of the patients at a distance of 3.0 cm, forming 45 channels (purple numbers). Detecting areas mainly cover DLPFC (BA 9, 46; yellow), VLPFC (BA 44, 45, 47; pink), and FPoC (BA 10, 11, 47; orange). BA, Brodmann's area; DLPFC, dorsolateral prefrontal cortex; VLPFC, ventrolateral prefrontal cortex; FPoC, frontal pole cortex.

During the multi-cognitive tasks, the relative concentration fluctuations of [oxy-Hb] and [deoxy-Hb] are recorded to reflect different mental disorders from a 45-channel fNIRS system (Foire-3000, Shimadzu Corporation, Japan) (see Figure 1).

**Figure 2:**
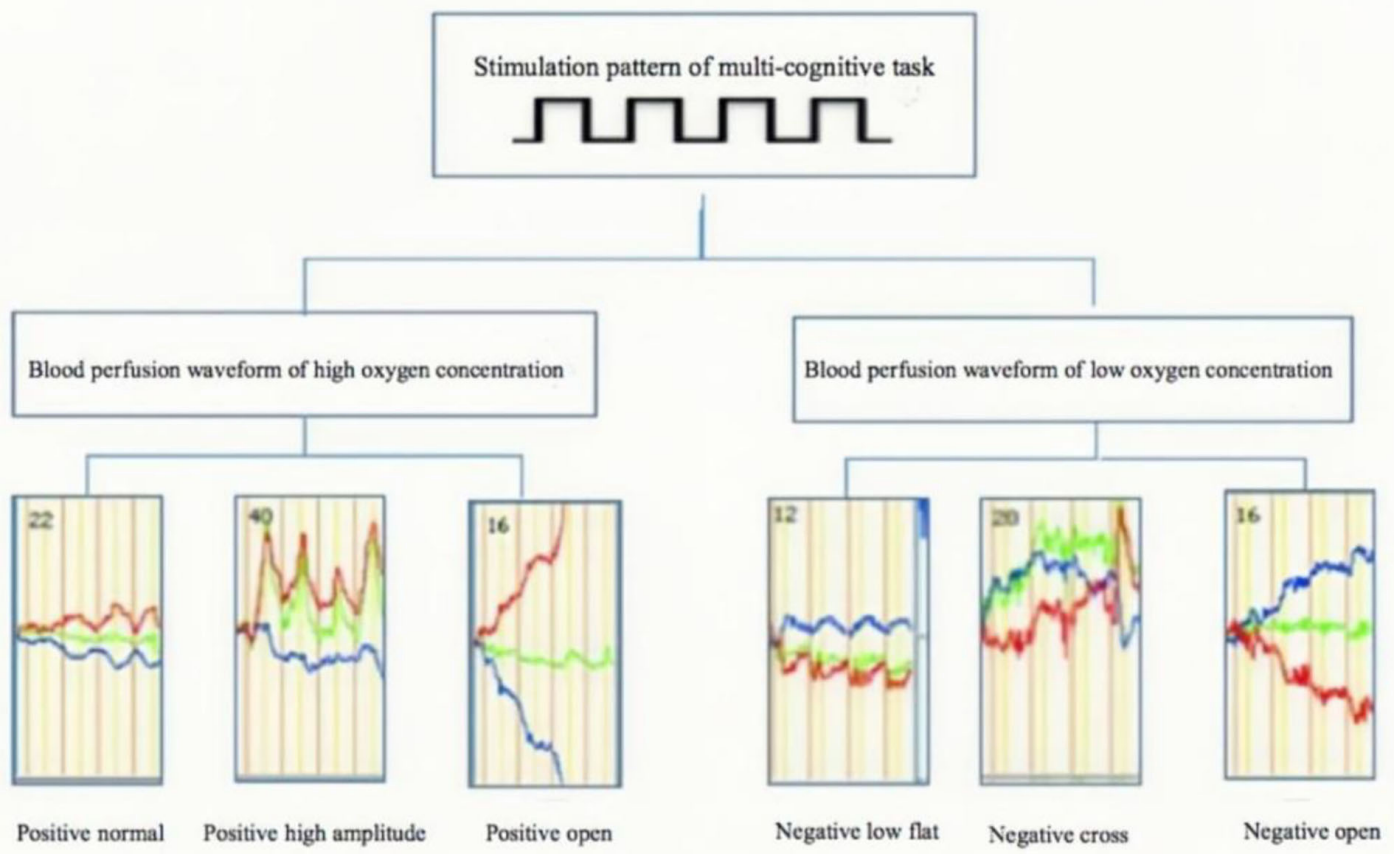
Hyperbolic waveform of multi-channel [oxy-Hb] and [deoxy-Hb] blood perfusion.

The symptoms (states) are identified and analyzed in the way of full-channel waveform, phase changes, and waveform composition ratios, and six typical waveforms (see Figure 2) with clinical significance are summarized to correlate with the typical symptom among various mental disorders (17). It has helped in the diagnosis of bipolar disorder and unipolar depression (15), major depressive disorder and generalized anxiety disorder (16), schizophrenia (28), and other mental disorders.

## Functions of the prefrontal working memory network

The aim is to analyze functional abnormalities in the prefrontal cortex with fNIRS using various paradigms in psychiatry, with (letter) verbal fluency task as the primary task in the clinical diagnostic setting [e.g., (29)] and other supplementary tasks such as emotional facial recognition task [e.g., (30)], working memory tasks [e.g., (31)], and emotional words task [e.g., (32)]. However, the lack of theoretical explanations for these functional imaging atlases has greatly hindered the application of fNIRS in psychiatry. The theoretical linkage between the fNIRS data showing neurovascular coupling and the patterns of the working memory network is unclear.

**Figure 3:**
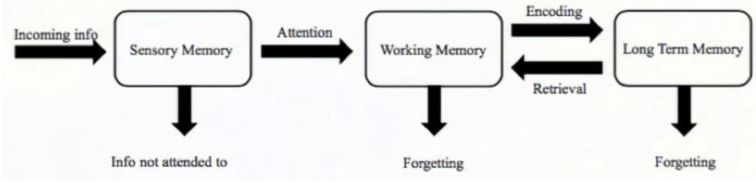
Information processing model.

One of the primary functions of the prefrontal lobe is executive function. In empirical and theoretical papers, primary factors underlying executive functions are: (a) inhibition and switching (33–35), (b) working memory (34, 36–40), and (c) sustained and selective attention (37, 39–41). Originating from Baddeley and Hitch (42), there are several models of working memory [e.g., (43, 44)]. One common feature shared is the limited capacity resource of working memory that some theorists relate to attention [e.g., (44, 45)]. It is clear that the prefrontal function is closely related to attention, working memory, and executive function. Based on the memory model of information processing (46), which is the information flow of stimulus to memory (see Figure 3), the potential problems of working memory network are presented among patients of psychiatric disorders with fNIRS. Based on the practical diagnosis using multi-cognitive tasks and theoretical studies on the prefrontal cortex, we propose that the brain imaging of the prefrontal cortex in alternating stimulus modes of resting and task modes reflects the joint working mode of the three memory networks: the attentional working memory primary coordination network, the perceptual content working memory secondary network, and the emotional-behavioral working memory executive network.

### Attentional working memory primary coordination network

In the neural network, the attentional working memory network is regarded as the main coordination network due to its importance. Attention is the taking possession by the mind, in clear and vivid form, of one out of what seem several simultaneously possible objects or trains of thought with the essence of focalization and concentration of consciousness. It implies withdrawal from some things in order to deal effectively with others [(47), pp. 403–404)]. Broadbent's (48) attention filter theory states that stimuli that are given notice and attention could enter working memory for further information processing. In the information processing model, attention serves as a filter.

An important function of prefrontal DLPFC (18) is to guide thinking and attention in a top-down pattern. Prefrontal cortex deficits can lead to attention disorders, including attentional impairment, distraction, and supervisory attention disorder, difficulty in shifting attentional focus, selective attention disorder, and decreased arousal. According to the pool-of-resources theory, we regard attention more as a resource pool, which can be flexibly allocated in competing tasks. In patients with DLPFC impairment, the distribution of attention is unorganized and uncontrollable.

Attention plays an important role in multi-cognitive tasks. Through the four tasks in the multi-cognitive tasks, attention is distributed and adjusted according to the timing and task requirements, reflecting its status in the working memory network. Patients need to pay attention to the multi-cognitive tasks which take 11.5 mins in total. The varied requirements and cognitive contents lead to the adapted allocation and consumption of attentional resources. Attention should be allocated flexibly to the four different cognitive tasks. For example, in the emotional picture identification task, patients need to focus on the picture, and judge its emotional valency according to the emotional effect of the visual stimulation presented. While in the main (category) verbal fluency task, patients need to retrieve words that belong to the same category, which requires the involvement of language processing. The distribution of attention should be automatic and adjusted flexibly according to the scene in healthy controls with little cognitive consumption, thus showing a relatively flat waveform. However, the recorded waveforms by fNIRS in multiple cognitive tasks are messy and of great fluctuation in patients with attention deficits. It is shown that attention engagement and its flexible allocation are required to ensure smooth performance on multiple cognitive tasks. Besides, the attentional working memory primary coordination network influences the subsequent perceptual content working memory secondary network and the emotional-behavioral working memory executive network.

### Perceptual content working memory secondary network

The central executive function of the prefrontal cortex is closely related to working memory. Working memory is the ability to keep in mind an event that has just occurred, or bring to mind information from long-term storage, and use this representational knowledge to regulate behavior, thought, and emotion. During information processing, only the stimulus given attention could enter the working memory for further processing. We believe that with allocated attention, the perceptual content working memory secondary network is the working memory network responsible for cognition assessment and thinking regulation. It can be evaluated and analyzed through two main tasks in multiple cognitive tasks, namely (category) verbal fluency task and the setting-based sentence fluency task.

Take the most common working memory model as an example, Baddeley and Hitch's (42) model divided working memory into the central executive and the slave system. The central executive is the overall controller to coordinating among different tasks. The slave system includes a phonological loop and visuospatial sketchpad. The phonological loop holds the speech-based information for rehearsal. Visuospatial sketchpad holds visual-spatial information not only for its initial processing but also when it is later retrieved from long-term memory. The final element of the model, the episodic buffer (49), is responsible for the integration of information from different components of both working memory and long-term memory.

The difference between auditory and visual stimulus processing in the model demonstrates that modality plays its function, as shown in the division of phonological loop and visual-spatial sketchpad. In multi-cognitive tasks, task instructions are all presented with both visual and auditory modalities. Pictures are presented in both the emotional picture identification task and the setting-based sentence fluency task to activate the visuospatial sketchpad. The setting-based sentence fluency task requires a description of the picture and triggered feelings, which requires the integration of working memory and long-term memory information, thus activating episodic buffer. Whereas in the (category) verbal fluency task, vocabularies are presented on the computer screen along with its auditory speech sounds, activating mostly the language-related factor, the phonological loop. The multiple cognitive tasks cover multiple modalities, auditory and visual, verbal and pictorial, activating working memory to a larger extent.

Besides, the differences of perceptual contents in multiple cognitive tasks lead to various working memory demands, i.e., the cognitive resources varying among different tasks. The emotional picture task involves mostly instantaneous memory, in which judgments about the emotion valency pictures are momentarily presented. The finger-tapping task is more likely to reflect somatization control with finer regulation. (Category) Verbal fluency tasks and setting-based sentence fluency tasks both involve language processing, which requires the involvement of both working memory and long-term memory. In (category) verbal fluency task, patients need to activate and retain the presented word such as “vegetable” in mind, that is, short-term memory needed to maintain current task requirements, and then extract the required words through word association network to seek the subcategory of the word “vegetable,” which activates the semantic memory in the long-term memory. In the setting-based sentence fluency task, pictures enter the short-term memory and activate the visuospatial sketchpad of working memory. The integration of the relevant information shown in the picture to show the relevant information and the emotions aroused by the long-term schemas is needed. Especially, the cognitive resource demand for sentence production in setting-based sentence fluency tasks is much higher than that of word retrieval in (category) verbal fluency tasks. Following Levelt (50), we may distinguish four stages of production: conceptualizing, formulating, articulating, and self-monitoring. We conceptualize what we wish to communicate, formulate this thought into a linguistic plan, execute the plan through the muscles in the speech system, and monitor our speech. The setting-based sentence fluency task at the sentence production level is more difficult than the (category) verbal fluency task at the vocabulary retrieval level. To some degree, the difficult differences in perceptual content among tasks of picture judgment, movement imitation, and language processing enlarge the scope of cognitive resource consumption, which helps to improve the discrimination validity.

### Emotional-behavioral working memory executive network

Another important function of the prefrontal cortex is the control and regulation of emotions (51). According to Arnsten (18), patients with psychiatric disorders become uncontrolled, reacting in a reflexive and rapid manner, mainly due to the decreased prefrontal function leading to the dominance of emotions taken over by amygdala. Patients pay attention to the most salient stimulus and lack the ability to control their attention. Therefore, we believe that the executive functioning of the brain under different perceptual content tasks is regulated by emotion.

It is widely accepted that the valence of a word (neutral, positive, or negative) influences lexical processing. The model of motivated attention and affective states (52) proposes that emotional stimuli, regardless of polarity, capture attention to a greater extent than neutral stimuli due to the survival-related salience which both positive (e.g., food) and negative (e.g., threat) stimuli convey. In order to minimize the emotional effects of words in (category) verbal fluency task, neutral category words are chosen in the (category) verbal fluency task, functioning as a benchmark of emotions. Thus, the neutral characteristic of (category) verbal fluency task could reflect the homeostasis of subjects' emotions. Both the emotional picture identification task and the setting-based sentence fluency task use pictures with emotional valence as a stimulus to examine patients' responses to and processing of emotional information. The emotional picture identification task is the decision of overall feeling toward pictures with positive, negative, and neutral valency. Whereas the setting-based sentence fluency task involves only pictures with negative valency, along with higher order thinking. Compared with the emotional picture identification task, the setting-based sentence fluency task has a higher cognitive load as language production is required (see section Emotional-behavioral working memory executive network), through which the influence of cognitive burden on emotion regulation and processing is reflected. By comparing the two tasks, how participants cope with more difficult tasks and how their perception of emotions under pressure could be manifested. Emotional instability and more depression-anxiety waveforms are seen in setting-based sentence fluency tasks with emotion arousal compared to emotional picture identification tasks as well as (category) verbal fluency tasks.

## Discussion and future directions

Working memory networks, as a central element in cognition, are of great significance. Language is a verbal behavior of human intelligence, in which its underlying cognitive and psychological mechanisms are inseparable from working memory. It is pointed out that research should be based on cognition and psychology, seeking its behavioral manifestations outward and its neural basis inward (53). Based on clinical diagnosis and the summarized six classical waveforms, we further delineate the traditional classical cognitive and psychological mechanism—working memory. The investigation of patients' attention, their performance on various perceptual contents, and emotional behavior executive functions from a cognitive perspective will facilitate the study of relevant neural mechanisms, which is of great significance for the development of cognitive science and phenotypic markers in medicine. The characteristics of the working memory network are indirectly represented through fNIRS by detecting the relative concentration changes in [oxy-Hb] and [deoxy-Hb], which contribute to a further neurological understanding of the working mode and characteristics of the prefrontal working memory network.

It is found that prefrontal functional connections during cognitive tasks are related to the neurovascular coupling mechanism and the effective utilization pattern of energy (oxygen) during cerebral nerve activity (54–56). The coordinated operation of the working memory network is based on the efficient use of energy by neurotransmitters and neurovascular coupling mechanisms, in which six typical waveforms in multiple cognitive tasks are extracted (see Figure 2). In normal people or people who recovered from anxiety disorders, it is observed that the red line of [oxy-Hb] relative concentration curve is on the top, and the curve of [deoxy-Hb] relative concentration is on the bottom, with a parallel hyperbola. During the onset of depression, attention is weakened and the degree of neuronal activation is reduced. In the symptom mapping classification compared to the mapping of normal people, the phase is of inverted parallel shape, in which the blue line [deoxy-Hb] is at the top and the red line [oxy-Hb] at the bottom, showing an inverse parallel shape, representing the inefficient use of oxygen at this locus.

In the anxiety state, the brain is too sensitive to external information, with increased attention and hyperactivity, which is reflected by the high peaks in the symptom classification mapping of multiple cognitive tasks. In the compulsion state, attention is enhanced with the brain repeatedly excited on the same task; thus, the brain continues to provide energy to this region. The increased attention to a stimulus will lead to the hyperexcitation of neurons, which is manifested in the continuous increase of [oxy-Hb] concentration. On the symptom classification mapping, the superposition of waveforms is seen, with an open hyperbola. The open positive phase indicates that the relative concentration of [oxy-Hb] at this site is higher than that of [deoxy-Hb]. The open negative phase indicates that the relative concentration of [deoxy-Hb] at this site is higher than that of [oxy-Hb]. Both of which may be a localized persistent pathological state. In adolescent children with attention deficit disorder or bipolar disorder with mixed episodes of inattention, and in mixed states of anxiety and depression, crossover disordered waveforms can be observed, i.e., the relative concentration curve of [deoxy-Hb] and the relative concentration curve of [oxy-Hb] are crossed, and the hyperbolic amplitude increases significantly increased and the waveforms are disordered.

From the perspective of neural mechanism, almost all the current fNIRS research only analyzes the changes in the relative concentration of [oxy-Hb], and little is known about the effect of [deoxy-Hb] on the neurovascular coupling mechanism. Neurons, vascular smooth muscle cells/pericytes, and astrocytes are involved in neurovascular coupling. The destruction of physiological structures such as the peritubular space, blood–brain barrier, and humoral factors such as Ang can cause neurovascular coupling dysfunction. Part of the action mechanisms of different types of neurons, key neurotransmitters, receptors, and ions in related pathways are closely related to the maintenance of brain homeostasis because the neurovascular coupling mechanism widely exists in the brain and presents the characteristics of multi-structural participation and multi-channel regulation (57).

It is generally believed that neurovascular coupling is only a marker of neuronal activation, with an amplitude proportional to the degree of neuronal activation, and changes in neurovascular coupling under disease are rarely considered. Csipo et al. (58) found that the fNIRS approach can distinguish the representation of the neurovascular coupling mechanism in cognitive tasks of different difficulties. For example, in pathological states such as aging and cognitive decline, decreased clearance of metabolites in peritubular space may be one of the important pathological mechanisms of neurovascular coupling dysfunction (59). Both psychiatry and neurology need to pay attention to the changes in phase, amplitude, and waveform of the relative concentration curve of [oxy-Hb] and [deoxy-Hb] in the neurovascular coupling mechanism of the prefrontal working memory network represented by fNIRS.

Multiple cognitive tasks analyze fNIRS imaging based on neurovascular coupling mechanism through cognitive tasks with different difficulties and divide the working memory network into three cognitive-related sub-networks for the first time. The near-infrared detection of multiple cognitive tasks presents the vascular coupling mechanism of the prefrontal neural network and neural working loops. Our articles (15–17) support the idea that the intracellular signal pathway network is blocked under stress as proposed by Arnsten (18). Aided by fNIRS, the sub-systems of the working memory network are represented, namely the attentional working memory primary coordination network, the perceptual content working memory secondary network, and the emotional-behavior working memory executive network.

The subdivision of prefrontal working memory networks based on neurovascular coupling offers a novel insight into the analysis of fNIRS imaging representations, and also provides a direction for drug evaluation. This study may help expand the clinical application of fNIRS through the informed diagnosis of mental disorders and provide physicians with an objective measure that may eventually be used to personalize treatment. With the development of brain function imaging and imaging analysis technology, such as dynamic brain atlas, a more accurate and refined representation of the working memory network will be represented.

## Data availability statement

The original contributions presented in the study are included in the article/supplementary material, further inquiries can be directed to the corresponding author.

## Author contributions

YR drafted the manuscript according to PL's clinical experience and was inspired by GC's psychological accounts. PL and YR discussed the theoretical framework and went through several minor changes. PL, GC, XZ, KF, and CY approved the final version of the manuscript with their guidance and comments on the article. All authors contributed to the article and approved the submitted version.

## Conflict of interest

The authors declare that the research was conducted in the absence of any commercial or financial relationships that could be construed as a potential conflict of interest.

## Publisher's note

All claims expressed in this article are solely those of the authors and do not necessarily represent those of their affiliated organizations, or those of the publisher, the editors and the reviewers. Any product that may be evaluated in this article, or claim that may be made by its manufacturer, is not guaranteed or endorsed by the publisher.
